# Inhibition of Nitric Oxide (NO) Production in Lipopolysaccharide (LPS)-Activated Murine Macrophage RAW 264.7 Cells by the Norsesterterpene Peroxide, Epimuqubilin A

**DOI:** 10.3390/md8030429

**Published:** 2010-03-01

**Authors:** Sarot Cheenpracha, Eun-Jung Park, Bahman Rostama, John M. Pezzuto, Leng Chee Chang

**Affiliations:** Department of Pharmaceutical Sciences, College of Pharmacy, University of Hawaii Hilo, 34 Rainbow Drive, Hilo, HI, 96720, USA; E-Mails: cheenpracha@gmail.com (S.C.); pinosylvin@hotmail.com (E.-J.P.); rostama@hawaii.edu (B.R.); pezzuto@hawaii.edu (J.M.P.)

**Keywords:** nitric oxide production, norsesterterpene peroxide, Latrunculia sp., RAW 264.7 Cells

## Abstract

Seven norsesterterpene peroxides: epimuqubilin A (**1**), muqubilone B (**2**), unnamed cyclic peroxide ester (**3**), epimuqubilin B (**4**), sigmosceptrellin A methyl ester (**5**), sigmosceptrellin A (**6**), and sigmosceptrellin B methyl ester (**7**), isolated from the marine sponge *Latrunculia* sp., were examined with regard to their effects on nitric oxide (NO) production in lipopolysaccharide (LPS)-activated murine macrophage RAW 264.7 cells. The results indicated epimuqubilin A (**1**) possessed potent NO inhibitory activity against lipopolysaccharide (LPS)-induced nitric oxide release with an IC_50_ value of 7.4 μM, a level three times greater than the positive control, L-N^G^-monomethyl arginine citrate, followed by **6** (sigmosceptrellin A, IC_50_ = 9.9 μM), whereas other compounds exhibited only modest activity (Table 1). These compounds did not show appreciable cytotoxicity at their IC_50_ values for NO–inhibitory activity. The structure–activity upon NO inhibition could be summarized as follows: (1) a monocyclic carbon skeleton framework was essential for activity, (2) free acids gave higher activity, (3) the orientation of H_3_-22 with an equatorial position increased activity, and (4) a bicyclic structure reduced activity. This is the first report of a norsesterterpene peroxide with NO–inhibitory activity. In addition, compounds **1**–**7** were also evaluated for their inhibitory activities in the yeast glycogen synthase kinase-3β assay. In summary, several norsesterterpene peroxides showed novel biological activities of inhibition in NO production, suggesting that these might provide leads for anti-inflammatory or cancer chemopreventive agents.

## 1. Introduction

Sesterterpenes isolated from marine organisms are often modified by the loss or addition of one or more carbon units. Norsesterterpene peroxides are characterized with a 2-substituted propionic acid or methyl propionate group attached to a 1,2-dioxane ring at the C-3 position.

Marine sponges of the Latrunculiidae family, which include the genera *Sigmosceptrella* [1,2], *Latrunculia* [3,4] and *Diacarnus* [5,6], have proven to be a rich source of norterpene cyclic peroxides, a novel class of bioactive marine metabolites. Sponges of the genus *Latrunculia* have a variety of the biosynthetically diverse secondary metabolites. The Red Sea sponge *Latrunculia magnifica* has been shown to contain macrocyclic thiazoles [7], while New Zealand, Australian, and Argentine species of *Latrunculia* have been shown to contain a variety of peptides and nitrogen-containing metabolites [8–12]. On the other hand, Australian *Latrunculia* species have been shown to be a rich source of novel norterpene acids and peroxides [3,4,13]. Interest has been focused on terpene peroxides as they frequently posses a vast array of biological activities such as ichthyotoxicity [1,2], antimicrobial [4], antitumor [6], cytotoxicity [5,14], sea urchin egg cell division inhibitory properties [15], antiviral [16], antitoxoplasmodic [16], and antimalarial activities [16]. In spite of the number of studies that have been performed, there has been no investigation of the anti-inflammatory activity of the norterpenoids.

Inflammation is the protective process of microcirculation in the host against a wide range of injuries caused by physical force, irradiation, extreme temperature, irritants and mostly infectious pathogens. However, dysregulated and perpetual inflammation may cause various pathophysiological conditions including gastritis, esophagitis, hepatitis, atherosclerosis, as well as cancer [17,18]. Macrophages are involved in chronic inflammation by producing various inflammatory mediators including cytokines, chemokines, interferon, colony-stimulating factors, lysozymes, proteases, growth factors, eicosanoids, and nitric oxide (NO) [19,20]. Among these, NO is excessively generated by one of pro-inflammatory enzymes, iNOS, and consequently results in diverse diseases and including asthma, arthritis, multiple sclerosis, colitis, psoriasis, neurodegenerative disorders, tumor development and transplant rejection of septic shock [21]. Therefore, it is of interest to find new inhibitors of NO production.

Glycogen synthase kinase-3β (GSK-3β) is a serine/threonine kinase found to be ubiquitous distributed throughout the body and to play a central role in many cellular and physiological events [22]. Inhibition of the GSK-3β pathway has emerged as a potential therapeutic approach for a number of pathologies [23]. In our continuing efforts to identify new protein kinase inhibitors for potential use as anticancer agents, a yeast glycogen synthase kinase-3β assay was adopted, to rapidly screen and identify candidate compounds that target serine/threonine and/or tyrosine kinases activities.

The crude organic extract from *Latrunculia* sp. showed an inhibition of nitric oxide (NO) production in lipopolysaccharide (LPS)-stimulated RAW 264.7 cells with an IC_50_ value of 16.6 μg/mL. Further fractionation led to the isolation of seven norsesterterpene cyclic peroxides including epimuqubilin A (**1**) [14], muqubilone B (**2**) [24], unnamed cyclic peroxide ester (**3**) [25], epimuqubilin B (**4**) [14], sigmosceptrellin A methyl ester (**5**) [1,18], sigmosceptrellin A (**6**) [25], and sigmosceptrellin B methyl ester (**7**) [25]. Herein we report the isolation, structure elucidation, and biological activities of these compounds. Compounds **1**–**7** were also evaluated for their inhibitory activity in the yeast GSK-3β assay.

## 2. Results and Discussion

**Figure 1:**
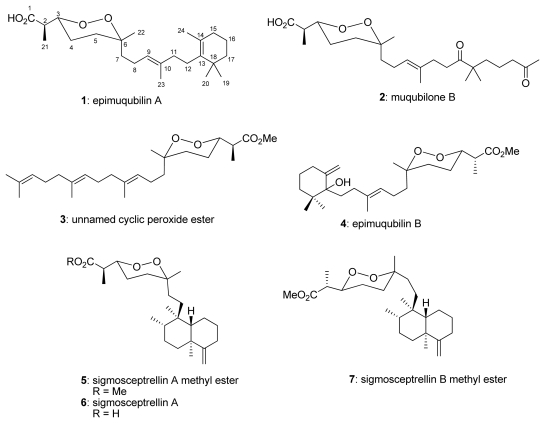
Structures of compounds 1–7.

Seven cyclic peroxides (Figure 1) were isolated from an organic extract of *Latrunculia* sp. C006879 and were tested for their NO inhibitory activity using RAW 264.7 cells. The results indicated epimuqubilin A (**1**) possessed potent NO inhibitory activity against lipopolysaccharide (LPS)-induced nitric oxide release with an IC_50_ value of 7.4 μM, followed by **6** (sigmosceptrellin A, IC_50_ = 9.9 μM), whereas other compounds exhibited activity with the IC_50_ values in the range of 23.8–65.7 μM. Sigmosceptrellin A methyl ester (**5**) was inactive in the NO inhibitory assay. The potency of **1** was comparable to that of L-N^G^-monomethyl arginine citrate, a positive control (IC_50_ = 25.5 μM). All compounds showed no cytotoxicity at their IC_50_ values for NO inhibitory activity. Structure–activity relationships of these compounds against NO release indicated the following: (1) a monocyclic carbon skeleton framework was essential for activity as observed in **1** (IC_50_ = 7.4 μM) *versus* **2** (IC_50_ = 23.8 μM), (2) a free acid gave higher activity than an ester, as observed in **6** (IC_50_ = 9.9 μM) versus **5** (IC_50_ >100 μM), (3) the orientation of H_3_-22 at an equatorial position increased activity, as observed in **7** (IC_50_ = 65.7 μM) versus **5** (IC_50_ > 100 μM), and (4) a bicyclic ring reduced activity, as observed in **6** (IC_50_ = 9.9 μM) versus **1** (IC_50_ = 7.4 μM).

In addition, to determine whether the inhibitory effects of compounds on NO production were due to specific pharmacological activities, or non-specific cell cytotoxicity leading to false positive results, RAW 264.7 cell viability was determined by using the sulforhodamine B (SRB) protein staining method. Compounds **1** and **6** mediated a modest cytototoxic response, with 36.1 and 43.0% percent survival, respectively, at the highest concentration tested (20 μg/mL; 51.0 μM). Cytotoxic effects diminished with lower concentrations. Notably, at a concentration of 12.8 μM (above the IC_50_ values of compounds **1** and **6**), no significant cytotoxic effects were observed. Further experiments will be performed to determine whether epimuqubilin A inhibits nitrite production via suppression of iNOS expression in the levels of protein.

Compounds **1**–**7** were also evaluated for their inhibitory activities in the yeast GSK-3β assay at both 25 and 37°C. Compounds **1**, **4**, and **6** exhibited significant inhibitory activities against yeast GSK-3β plates (Table 1) and gave 10–13 mm zones of inhibition (ZOI) at 40 μg/disk. All other isolated compounds were inactive. The clear ZOI at 37°C and no ZOI at 25°C indicated the inhibition of GSK-3β pathway. Compounds **1**, **4** and **6** exhibited potent inhibitory activity and gave 8 mm clear ZOI at 20 μg/disk at 37°C and no ZOI at 25°C, indicating that these compounds inhibited GSK-3β pathway. Compound **6** appeared to be more potent as compared with **1**, indicating that the presence of the bicyclic moiety was essential for activity.

In this study, we found that epimuqubilin A (**1**) possessed potent NO production in LPS-stimulated RAW 264.7 cells and sigmosceptrellin A (**6**) exhibited significant of the selective GSK-3β pathway. This is the first report of the norsesterterpene peroxides with the NO–inhibitory activity.

Compounds **1**, **2**, **5**, and **6** have been reported in treatment of human African trypanosomiasis against *Trypanosoma brucei* with IC_50_ values in the range of 0.2–2.0 μg/mL [25]. Compound **4** was isolated from the sponge *Diacarnus megaspinorhabdosa* and exhibited cytotoxic [5] and antimicrobial activities [25]. As described in the literatures, it was also observed that insertion of a prenyl unit in the central portion of the molecule made norsesterterpenes cyclic peroxides (compounds **1**–**7**) more active than their norterpene congeners (diacarperoxide A-C) [5]. It has been reported previously that a longer side chain allowed the norsesterterpenes to cross cell membranes (lipophilicity effect) and afforded a better fit to the receptor (side effect) [26]. In another study, the cytotoxic and antiparasitic properties for 1,2-dioxane and furan ketides, isolated from the sponge *Plakortis angulospiculatus* [27], were compared. These results showed that both heterocyclic ring displayed modest cytotoxicity, while one of the 1,2-dioxane structures potently inhibited *Leishmania chagasi* and *Trypanosoma cruzi*.

## 3. Experimental Section

### 3.1. Test Compounds

The sponge *Latrunculia* sp. (C006879) was collected at a depth of 15 m in the Indo-West Pacific (Indo-Polynesian), on September 6, 1990. The sponge was frozen immediately upon collection and shipped to NCI (National Cancer Institute, Frederick, MD). After aqueous extraction of the frozen soft corals at 4°C, the extracts were lyophilized and extracted sequentially with CH_2_Cl_2_-MeOH (1:1) and MeOH. The combined organic extracts were evaporated *in vacuo* and stored at −30°C. A voucher specimen was deposited at the National Museum of Natural History, Smithsonian Institution, Washington, D.C.

A crude organic extract (3.0 g), provided by the NCI Natural Products Open Repository collection, exhibited NO inhibitory activity with an IC_50_ value of 16.6 μg/mL. This extract was chromatographed on silica gel (150 g) using CC eluting with the stepwise with hexanes–EtOAc (1:9 → 0:10, each 200 mL), and EtOAc–MeOH (1:9 → 1:1, each 200 mL), respectively to afford seven fractions (F1-F7). Fraction F4 (744.4 mg) was further purified by reversed-phase CC (60 g) eluting with MeOH-H_2_O (3:2 → 0:10, each 100 mL) to give six subfractions (F4a-F4f). Compound **1** (5.6 mg) was isolated from subfraction F4b (30.4 mg) by recrystallization from MeOH-CHCl_3_ (1:1). Repeated reversedphase CC (10 g) with MeOH–H_2_O (3:2, 350 mL) on subfraction F4d (50.5 mg) gave compounds **2** (3.2 mg), **3** (1.0 mg), **5** (5.8 mg) and **7** (3.6 mg). Subfractions F4f (19.2 mg) was subjected to RP-HPLC (10 *μ*m, 10 × 250 mm, Econosil C18, 2.0 mL/min) eluting isocratically with MeOH-H_2_O (97:3) to afford compound **4** (5.6 mg, *t*_R_ 24.6 min). Fraction F6 (377.1 mg) was purified by silica gel CC (10.0 g) eluting with acetone-hexanes (1:4 → 1:1, each 100 mL) to give compound **6** (2.2 mg).

*Epimuqubilin A* (**1**): Colorless viscous oil; [α]*_D_*^21^+55.9 (c 0.027, CHCl_3_); IR (film) ν_max_ 3450, 2925, 1710, 1578, 1320 cm^−1^; ESIMS *m*/*z* 393.30 [M + H]^+^.

*Muqubilone B* (**2**): Colorless viscous oil; [α]*_D_*^21^+52.5 (c 0.002, CHCl_3_); IR (film) ν_max_ 3422, 2953, 1700, 1498, 1321 cm^−1^; ESIMS *m*/*z* 425.30 [M + H]^+^.

*Unnamed cyclic peroxide ester* (**3**): Colorless viscous oil; [α]*_D_*^21^−57.8 (c 0.013, CHCl_3_); IR (film) ν_max_ 2967, 1670, 1530, 1134 cm^−1^; ESIMS *m*/*z* 407.32 [M + H]^+^.

*Epimuqubilin B* (**4**): Colorless viscous oil; [α]*_D_*^21^+ 15.8 (c 0.017, CHCl_3_); IR (film) ν_max_ 3445, 2929, 1685, 1540, 1130 cm^−1^; ESIMS *m*/*z* 423.31 [M + H]^+^.

*Sigmosceptrellin A methyl ester* (**5**): Colorless viscous oil; [α]*_D_*^21^+57.9 (c 0.001, CHCl_3_); IR (film) ν_max_ 2935, 1665, 1434, 1150 cm^−1^; ESIMS *m*/*z* 407.32 [M + H]^+^.

*Sigmosceptrellin A* (**6**): Colorless viscous oil; [α]*_D_*^21^+51.3 (c 0.027, CHCl_3_); IR (film) ν_max_ 3380, 2960, 1723, 1560, 1140 cm^−1^; ESIMS *m*/*z* 393.30 [M + H]^+^.

*Sigmosceptrellin B methyl ester* (**7**): Colorless viscous oil; [α]*_D_*^21^−55.4 (c 0.003, CHCl_3_); IR (film) ν_max_ 2960, 1660, 1560, 1150 cm^−1^; ESIMS *m*/*z* 407.32 [M + H]^+^.

### 3.2. Assay for NO inhibitory effect using RAW264.7 cells

The inhibitory effects of samples on NO production were evaluated in LPS-activated murine macrophage RAW 264.7 cells, using a method modified from that previously reported [28]. Briefly, RAW 264.7 cells were cultured in Dulbecco’s modified Eagle’s medium (DMEM) supplemented with penicillin G sodium (100 units/mL), streptomycin sulfate (100 μg/mL), amphotericin B (0.25 μg/mL), and 10% fetal bovine serum (FBS). The cells were seeded in 96-well culture plates with 1 × 10^5^ cells/well and incubated for 24 h at 37°C in a humidified atmosphere containing 5% CO_2_. The cells were treated with samples dissolved in phenol red-free DMEM for 30 min followed by 1 μg/mL of LPS treatment for 20 h. The amount of NO in the cultured medium was measured by the Griess reagent. The standard curve was created by using known concentrations of sodium nitrite, and the absorbance was measured at 540 nm. To evaluate the cytotoxic effect of samples in RAW 264.7 cells in the assay condition, SRB assay was performed. Briefly, after the fixation with 10% trichloroacetic acid (TCA), cells were stained with 0.4% SRB solution in 1% acetic acid followed by dissolving bound SRB in 10 mM Tris-buffer. The optical density was determined at 515 nm.

### 3.3. Glycogen synthase kinase-3β assay

Disruption of four yeast GSK-3 homologs (MCK1, MDS1, MRK1 and YOL128C) in *gsk-3* null mutant confers a temperature-sensitive phenotype (growth defect at 37ºC), that can be suppressed by the expression of cloned mammalian GSK-3β [29]. Inhibition of GSK-3β should therefore mimic the phenotype of the *gsk-3* null mutant, indicated by growth inhibition of transformant of *gsk-3* null with pKT10-GSK-3β only at 37ºC. A genetically modified yeast strain, H10075 with genotype {*MAT α his3 leu2 ura3 trp1 ade2 mck1::TRP1 mds1::HIS3 mrk1 yol128c::LEU2* [pKT10-GSK-3β], was grown in 5 mL of liquid media of SC minus uracil, shaking, at 37ºC for 2 days. Four hundred-μL of the yeast culture was added to 100 mL of SC minus uracil agar [0.67% yeast nitrogen base without amino acids and ammonium sulfate, 0.5% ammonium sulfate, 2% D-(+)-glucose anhydrate, 1 mL each of 0.03 mg/mL of adenine (hemisulfate salt), 0.03 mg/mL of L-tryptophan, 0.03 mg/mL of L-leucine, 0.03 mg/mL of L-histidine and 1.5% bacteriological agar, pH 5.6]. To perform the assay, compounds and extracts at a concentration of 80 μg per disk were dispensed on filter disks, and placed on plates containing yeast seeded into SC minus uracil media. The Petri dishes were incubated at both 25ºC and 37ºC for 72 hours. Three phenotypes are observed on yeast plates. A clear zone of inhibition (ZOI) at 37ºC and no ZOI at 25ºC indicate that the agent tested inhibits the GSK-3β pathway. The clear zones of inhibition at both 37 and 25ºC of equal width indicate that the agents are cytotoxic. Plates that have a negative phenotype are plates that have no observable ZOI. Active compounds were then tested at lower concentrations (40, 20, 10, 5 μg per disk). The assays were performed in duplicate. GSK-3β inhibitor (4-benzyl-2-methyl-1,2,4-thiadiazolidine-3,5-dione), and staurosporine were used as positive controls.

## Figures and Tables

**Table 1:** Biological activity of tested compounds on the NO inhibitory activity and the yeast glycogen synthase kinase-3β.

Compounds	NO IC_50_, μM)	Cytotoxic activity		Yeast GSK-3β^a^ (mm) at 37 °C(μg/disk)			

		% Survival^b^	IC_50_, μM	40	20	10	5
Epimuqubilin A (**1**)	7.4	36.1	37.1	10	9	na^c^	na
Muqubilone B (**2**)	23.8	99.0	-	na	-	-	-
Unnamed cyclic peroxide ester (**3**)	46.0	>100	-	nt^d^	-	-	-
Epimuqubilin B (**4**)	25.6	>100	-	10	8	na	na
Sigmosceptrellin A methyl ester (**5**)	>100	90.6	-	na	-	-	-
Sigmosceptrellin A (**6**)	9.9	43.0	42.7	13	11	10	8
Sigmosceptrellin B methyl ester (**7**)	65.7	>100	-	na	-	-	-
L-NMMA^e^	25.5	>100	-	-	-	-	-
TDZD-8^f^ at 37°C				15	11	10	9
at 25°C				12	10	9	8

a Diameter of disk alone is 7 mm. Stock solutions were prepared in either dimethyl sulfoxide (DMSO) or methanol (MeOH). No zones of inhibition were observed with DMSO or MeOH as negative controls. Active compounds were retested at lower concentrations (10–2.5 *μ*g/disk).

b % Survival was tested at 20 *μ*g/mL after 20 h incubation in RAW 264.7 cells.

c Not active.

d Not tested.

e L-N^G^-Monomethyl Arginine citrate (L-NMMA), non-selective inhibitor of NOS. Staurosporine gave a 13 mm zone of inhibition at 80 *μ*g/disk at 37°C and no ZOI at 25°C.

f 4-Benzyl-2-methyl-1,2,4-thiadiazolidine-3,5-dione, GSK-3β inhibitor.
